# Global Research Trends in Artificial Intelligence and Type 2 Diabetes Mellitus: A Bibliometric Perspective

**DOI:** 10.7759/cureus.88114

**Published:** 2025-07-16

**Authors:** Paul Anthony Camacho López, Maria G Latorre-Arevalo, Pablo Camacho-Naranjo, Silvia J Villabona-Florez

**Affiliations:** 1 Faculty of Medicine, Universidade de São Paulo (USP), São Paulo, BRA; 2 Department of Research, Fundación Oftalmológica de Santander (FOSCAL), Floridablanca, COL; 3 College of Medicine, Faculty of Health Sciences, Universidad Autónoma de Bucaramanga (UNAB), Bucaramanga, COL; 4 Faculty of Mechanical Engineering, Universidad Pontificia Bolivariana (UPB), Floridablanca, COL

**Keywords:** artificial intelligence, bibliometrics, bibliometrix, citnetexplorer, machine learning, type 2 diabetes mellitus, vosviewer

## Abstract

Artificial intelligence (AI) applied to type 2 diabetes mellitus (T2DM) is transforming the diagnosis and management of this chronic disease, posing a significant public health challenge. Despite recent advances, there remains a gap in the systematization of knowledge regarding AI and T2DM, as well as in the identification of trends and scientific collaborations in this field. This study aims to conduct a comprehensive bibliometric analysis of academic output on AI applied to T2DM, mapping the main actors, collaboration networks, and predominant research themes from 2000 to 2024. A bibliometric analysis was conducted using the Web of Science database, focusing on scientific topics related to AI applied to T2DM from 2000 to 2024. Bibliometric tools such as Bibliometrix, VOSviewer, and CitNetExplorer were utilized to examine publication patterns, co-citation networks, and keywords. The analysis included 1,454 original articles and 134 reviews, aiming to identify the most influential authors, institutions, and countries in the field. The analysis revealed a growth rate of 1.7%, with significant increases observed between 2020 and 2024. The research highlighted the use of AI for the detection of diabetic retinopathy and continuous glucose monitoring as the primary areas of publication. China (27.4%) and India (20.5%) lead scientific production and international collaborations in this field, reflecting the globalization of health research. This study provides an overview of the current state and future opportunities in AI research applied to T2DM. The findings are valuable for researchers, healthcare professionals, and academic institutions, fostering progress in AI and T2DM through collaborative and ethical strategies. This bibliometric analysis contributes to guiding the development of health research policies and optimizing the use of AI in managing T2DM.

## Introduction and background

Noncommunicable diseases (NCDs) constitute a global epidemic. In 2021, NCDs accounted for 38% of all deaths and 68% of the top 10 causes of death worldwide [1]. Four major conditions, i.e., cardiovascular diseases, cancers, chronic respiratory diseases, and diabetes, are responsible for nearly 80% of NCD-related deaths, primarily affecting low- and middle-income countries with limited access to quality healthcare [1-3]. In this context, diabetes mellitus (DM) has become one of the most impactful NCDs, particularly type 2 diabetes mellitus (T2DM), which accounts for 90% of diabetes cases worldwide [4]. T2DM not only poses a high risk of severe complications such as cardiovascular diseases and retinopathy but also places a significant economic burden on global healthcare systems [5]. In 2021, the International Diabetes Federation (IDF) estimated that 537 million people had T2DM, equivalent to 10.5% of the global population, with costs exceeding $966 billion [6,7].

Due to its chronic nature and the need for continuous monitoring, T2DM demands stable and effective diagnostic methods. Effective management not only prevents complications but also improves patients’ quality of life [8,9]. However, conventional methods face limitations in addressing the complexity of this disease [10]. In this context, artificial intelligence (AI) has emerged as an innovative tool, leveraging advanced algorithms for tasks such as diagnosis, complication prediction, and treatment personalization [11-13].

AI is based on two essential components, namely, extensive databases and a variety of algorithms. From these, various subtypes emerge with specialized functions and capabilities. Among them, machine learning (ML) stands out, encompassing several key subtypes such as artificial neural networks, deep neural networks, deep learning (DL), natural language processing, and computer vision. ML can operate through supervised models, which predict outcomes validated by experts, or unsupervised models, which identify patterns without direct human intervention [14].

Given the constant management and multidisciplinary approach required by T2DM, it has become a primary focus for AI applications in healthcare. AI tools enable data integration that facilitates the monitoring of clinical parameters and contributes to the early detection of complications, allowing for more informed and personalized medical decisions [15-17]. However, challenges remain for broader implementation in clinical practice, such as validation in diverse clinical settings and addressing ethical and data privacy concerns [18]. Research in AI applied to T2DM has grown significantly, reflecting increasing interest in technologies to optimize its management [19,20].

This study aims to conduct a comprehensive bibliometric analysis of scientific production related to AI applied to T2DM using the Web of Science (WoS) database between 2000 and 2024 [21]. Using tools such as Bibliometrix, VOSviewer, and CitNetExplorer, citation networks, co-citation, and keywords were analyzed to identify trends, international collaborations, and predominant research areas [22].

This approach enables the examination of the evolution of scientific production based on indicators such as country of origin, the most productive institutions and authors, as well as the most impactful journals and articles. This analysis provides a comprehensive overview of the current state of research on AI and T2DM, helping to identify achievements, challenges, and areas requiring further exploration. The bibliometric analysis highlights the contribution of AI in improving T2DM diagnosis and treatment, revealing an increase in global and multidisciplinary collaboration [23]. This methodology stands out as an essential resource for prioritizing future research and strengthening public health and clinical decision-making, promoting innovations that enhance patients’ quality of life.

Literature review

T2DM is one of the main global health challenges of the 21st century, especially because most people affected by the disease live in low- and middle-income countries. In these countries, T2DM accounts for approximately 1.5 million deaths annually [24]. DM is a metabolic disorder characterized by prolonged hyperglycemia. As a chronic disease, it carries a higher risk of developing various comorbidities, including cardiovascular complications, hypertension, depression, thyroid disorders, chronic obstructive pulmonary disease, Alzheimer’s disease, and diabetic retinopathy (DR) [25]. Traditionally, researchers have portrayed T2DM as a homogeneous disease; however, increasing evidence suggests its heterogeneity [10].

Early detection and prediction of diabetes are essential to reducing the risks associated with morbidity and mortality. However, prolonged and costly diagnostic processes in conventional healthcare systems have driven the incorporation of advanced technologies for predicting the disease [16,26]. Additionally, managing DM patients requires continuous clinical monitoring to control glucose levels and prevent complications. This management relies on a multidisciplinary approach involving endocrinology, podiatry, nutrition, nephrology, and ophthalmology. Further, DM demands active and continuous participation from patients due to the significant impact of diet and exercise on clinical outcomes, requiring various self-management strategies [27].

Technological advances in digital health, designed to improve the effectiveness and efficiency of healthcare, promise a better future in diabetes care. Telemedicine and mobile health (m-Health), defined as “the use of electronic information and communications technologies to provide and support health care when distance separates the participants” [28], facilitate diabetes management by providing remote care and personalized education to patients. Tools such as statistical analysis, ML, and DL are increasingly integrated into these technologies to improve disease prediction and monitoring [17].

The development and use of technological advancements aimed at assisting patients and clinical decision-making have transformed healthcare by enabling continuous health monitoring, improving diagnostic accuracy, and fostering a more personalized approach to treating various diseases. One of the key advancements in integrating technology into diabetes care is the incorporation of AI, which has shown promising results in the primary and secondary prevention of diabetes. AI-based methods employ computational algorithms to analyze complex data, detect patterns, and create predictive models that facilitate early diagnosis, individualized treatment, and effective management strategies for people with diabetes [15]. By integrating these technologies, healthcare professionals can make informed decisions tailored to the specific needs of each patient, thereby improving clinical outcomes [18].

AI-based methods are increasingly used across various domains of diabetes research. Reviews and bibliometric analyses have focused on areas such as diabetes management [19], disease prediction and its complications [20], and kidney disease [29], among others. This growth in global research on diabetes detection and treatment over recent decades presents a challenge for researchers to stay updated on the latest findings. Therefore, understanding future projections, identifying knowledge gaps, and analyzing key factors in diabetes detection are crucial [30].

Global research on diabetes detection and treatment has grown considerably in recent decades, making it challenging for researchers to remain current with the latest findings. Simultaneously, understanding future trends, identifying knowledge gaps, and focusing on key factors in diabetes detection are essential. Bibliometrics in health research is a quantitative analysis method that uses indicators such as the number of publications, citations, and h-index to evaluate the productivity, impact, and visibility of studies, journals, and authors. This tool helps identify trends, high-research areas, and knowledge gaps, which are crucial for policy development, funding, and collaboration in healthcare. Moreover, bibliometrics allows monitoring scientific progress and guiding future research, optimizing resources, and improving decision-making in public health and clinical settings [31]. Bibliometric analysis, a methodology for systematically and comprehensively examining the literature, is widely used for the qualitative and quantitative analysis of scientific publications [32]. This approach is applied in various medical fields, such as gynecology [33] and cardiology [34], to quickly identify trends and key research topics and evaluate the distribution of authors, countries/regions, and journals within a specific study area. It facilitates identifying potential directions and future developments in research. DM is no exception. In recent years, multiple bibliometric studies have focused on this condition. In 2022, Zhang et al. presented an analysis of global trends and hotspots in research on exercise as an intervention for diabetes. This study provided a clear view of the impact of exercise on diabetes management, underscoring its growing relevance in public health and suggesting future research directions, such as integrating technological approaches into intervention programs [23]. In 2023, He et al. explored the research landscape on diabetes-associated cognitive dysfunction (DACD), noting a steady increase in publications over the past 23 years. Key focal points included potential pathophysiological mechanisms and the effects of antidiabetic drugs on DACD [35]. In 2024, Ferdaus et al. analyzed the literature on diabetes detection and classification from 2000 to 2023, finding that the most frequently used terms were “machine learning” and “diabetic retinopathy” [36].

Hence, bibliometric analysis in AI research on T2DM offers a fundamental tool for understanding and guiding the evolution of this field. By identifying key trends, highly relevant areas, and knowledge gaps, bibliometrics not only directs resource prioritization and policy development but also fosters international and multidisciplinary collaboration. These analyses are essential for driving innovations in diabetes detection and management, enhancing intervention approaches such as exercise and advanced technologies to improve clinical outcomes and patients’ quality of life.

## Review

Methodology

This study is a bibliometric investigation aimed at understanding the development and perspective of scientific production on AI and T2DM. The research consisted of two steps: a visualized systematic review and a bibliometric analysis [37]. For the review, all publications available in the WoS database were considered.

The Web of Science Core Collection (WoSCC) provided by Clarivate (Philadelphia, PA, USA) offers a comprehensive platform for evaluating scientific metrics across various academic disciplines [38]. Its key features include structured data organization, thematic classification, citation analysis, a global focus, and scalability capabilities. It is widely recognized as the leading database in the field of bibliometric analysis [39].

Search Strategy and Criteria

The retrieval search strategy was conducted by topic, combining keywords with Boolean operators such as machine learning, artificial intelligence, deep learning, and support vector machine, among others. Topic searches involved a comprehensive exploration, including titles, abstracts, author keywords, and “keywords plus” generated by WoSCC (Appendices) [40]. The inclusion and exclusion criteria were as follows. Inclusion criteria: (1) articles written in English, Spanish, or Portuguese; (2) original research or review articles; (3) studies focused on T2DM as the main outcome; and (4) AI technologies. Exclusion criteria: conference abstracts, unpublished manuscripts, duplicate or retracted publications, editorials, letters, notes, book chapters, data files, and non-peer-reviewed sources. Additionally, studies lacking comprehensive analytical data were excluded.

Data Extraction

An initial sample of 3,108 documents was retrieved from the WoSCC for the period between 2000 and November 2, 2024. Articles were manually screened by two authors based on the abstract information. Any discrepancies between the reviewers were resolved by involving a third author to reach a consensus. As this is not a systematic review, full texts were assessed only in specific cases to determine inclusion.

Bibliometric Analysis

Literature records and cited reference data were downloaded in text format (.txt). After cleaning, the data were imported separately into bibliometric tools for internal structure analysis. The bibliometric tools used in the analysis included R software version 4.4.2, VOSviewer version 1.6.20 (Centre for Science and Technology Studies, Leiden University), and CitNetExplorer version 1.0.0 (Centre for Science and Technology Studies, Leiden University). Microsoft Excel Office (Microsoft Corp., Redmond, WA) was used to construct the citation map by country and analyze annual trends in publications and citations. The R software package Bibliometrix [41] was used via Biblioshiny to analyze the 1,588 publications, collaborations, journals, and keyword trends in the field. The growth rate of publications, research keywords, and publication patterns (countries, institutions, and journals) was calculated. The publication growth rate over time was determined using the following compound annual growth rate formula [42]: growth rate = ((number of publications in last year or number of publications in the first year)/(last year - first year) - 1) × 100.

VOSviewer was used for the bibliometric analysis of 1,588 articles, including countries, authors, institutions, or keywords, to visually analyze the co-occurrence networks of countries, institutions, authors, journals, and keywords, represented as nodes in the co-occurrence network [43]. The size of the nodes indicates the frequency of occurrence, and the connections between nodes are represented by link lines. Nodes are grouped into clusters with the same color, and link strength represents influence or centrality [44]. Bibliometric indicators were summarized as total publications (TPs) and total citations (TCs). The h-index was used to evaluate the scientific output and impact of researchers [45]. The multiple country publications (MCP) ratio was used to measure the level of international cooperation [46]. The total link strength (TLS) was used to evaluate the connections between institutions. Additionally, metrics such as the Journal Citation Indicator (JCI), SCImago Journal Rank (SJR), Cites per Document (three years), CiteScore, and Journal Impact Factor (JIF) were employed to assess the quality of publications [47].

CitNetExplorer was used to perform cluster analysis. These metrics highlighted the volume of publications over time, identifying the most influential articles and authors through citation analysis. The study also mapped co-authorship networks to reveal collaboration patterns among researchers and institutions, providing insights into the structure of the research community. Keyword analysis enabled the examination of research foci, emerging trends, and potential gaps in the existing literature, highlighting areas ripe for future exploration [48].

Results

**Figure 1:**
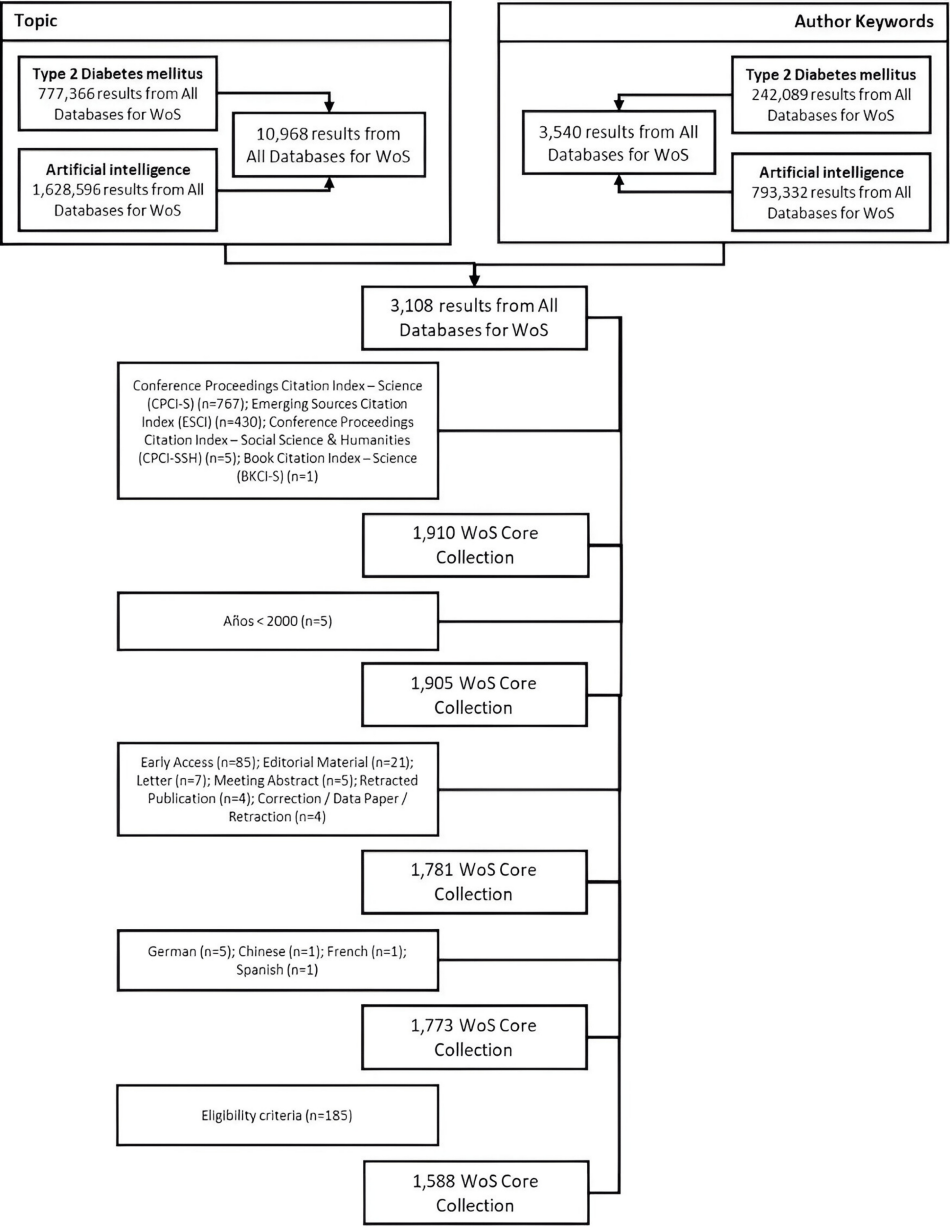
Flowchart of the search strategy in Web of Science.

Initially, 3,108 articles were identified in the WoSCC, with 61.45% (1,910) originating from the Science Citation Index Expanded (SCI-EXPANDED) and Social Sciences Citation Index (SSCI) databases. After excluding 137 articles due to study type, publication year, and language, and 185 due to thematic irrelevance, 1,588 publications (91.57% original articles and 8.43% reviews) from 2000 to 2024 were included in the bibliometric analysis (Figure 1).

Evolution of Scientific Production

**Figure 2:**
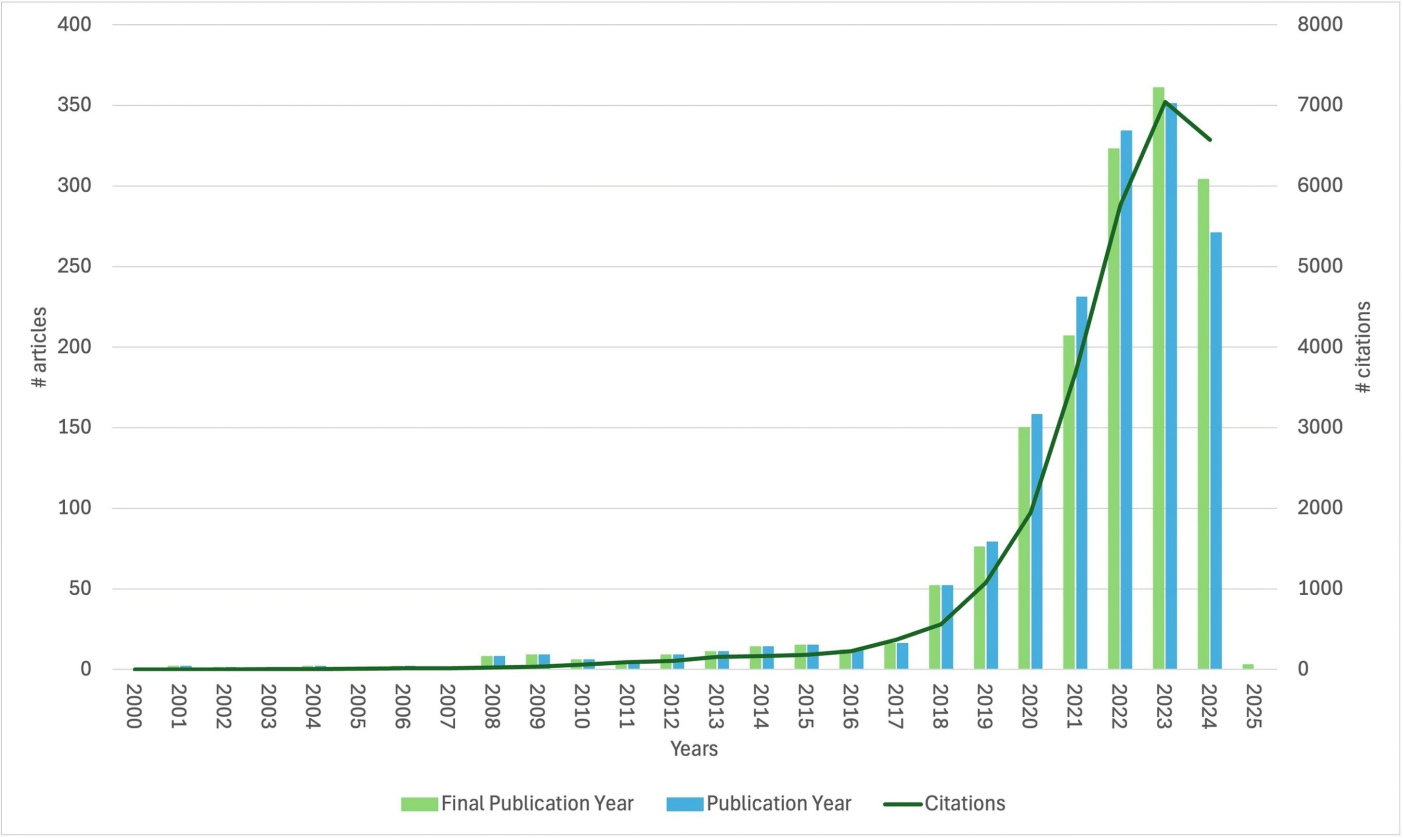
Trend in publications on artificial intelligence and type 2 diabetes mellitus (2000-2024).

Figure 2 illustrates the annual publication volume, reflecting the growing trend in this field of study. The evolution of scientific production can be divided into the following phases: (1) Initial stage (2000-2012): fewer than 10 publications per year; (2) steady ascending phase (2013-2017): a slow but consistent increase in publications, indicating growing interest in the subject; (3) early rapid development (2018-2019): the number of articles tripled compared to 2017; and (4) rapid growth (2020-2024): the annual publication volume rose sharply from 76 articles to a peak of 361 articles, with this growth expected to continue. Scientific production growth intensified during the 2020-2024 period, totaling 1,348 (84.89%) articles. Open-access articles accounted for 65.42% of the total, allowing unrestricted access to the content. The annual growth rate was 1.7%, with an average of 17.72 citations and 3,983 citations per article per year/document. The average age of the articles was 2.64 years.

Research Areas

**Figure 3:**
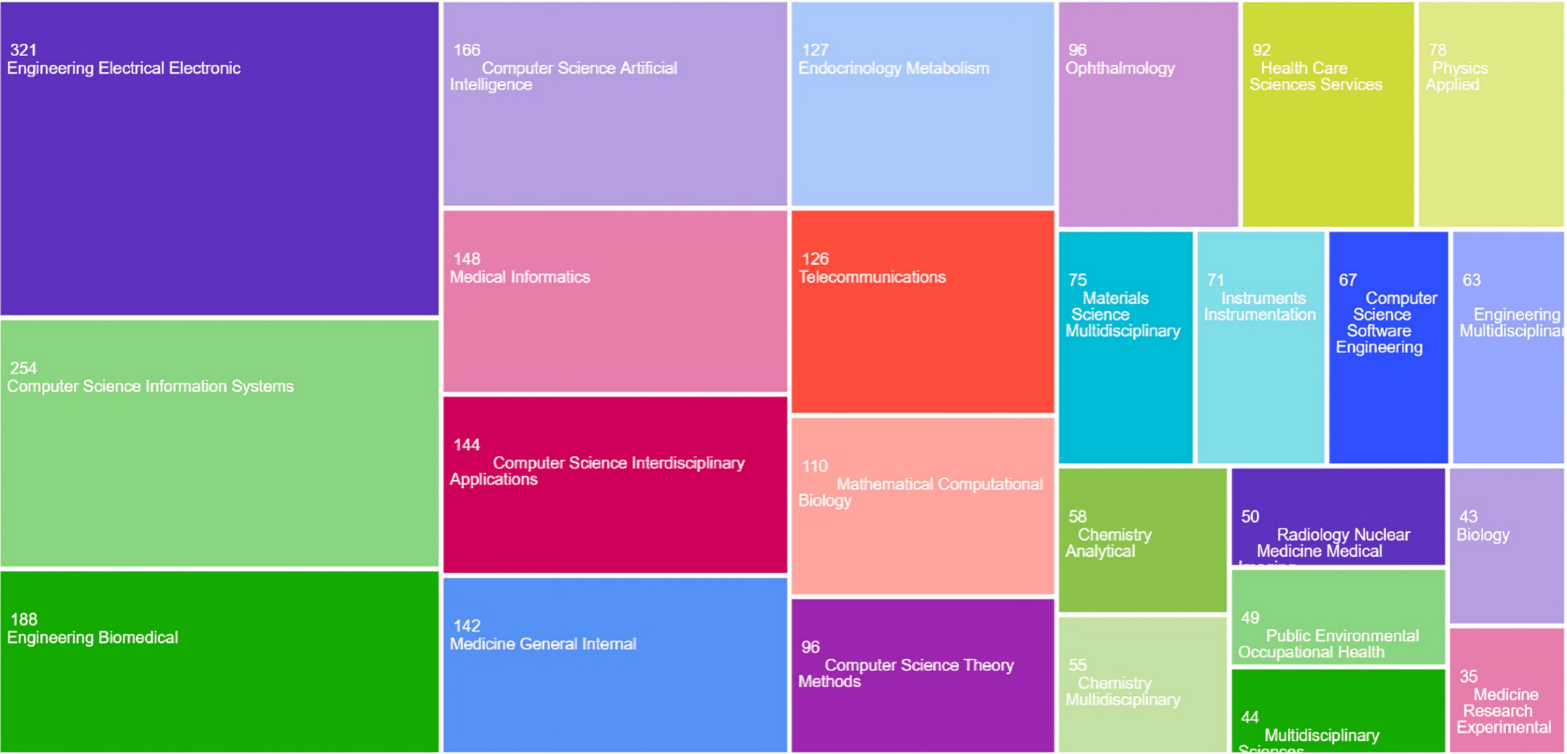
TreeMap Web of Science categories.

The distribution of articles revealed that AI applied to T2DM is correlated with and connects various fields of knowledge (Figure 3). The topics spanned from electrical and electronic engineering to endocrinology, ophthalmology, and AI-based computational methods. The five main categories were engineering electrical electronic (321 articles), computer science information systems (254 articles), engineering biomedical (188 articles), computer science artificial intelligence (166 articles), and medical informatics (148 articles). Electrical and electronic engineering stood out for its contributions to the development of devices for diagnosing, monitoring, and predicting glucose levels. In endocrinology and ophthalmology, AI focused on studying the natural history of the disease, particularly in diagnosing and predicting macular edema and DR.

Contribution by Countries

**Figure 4:**
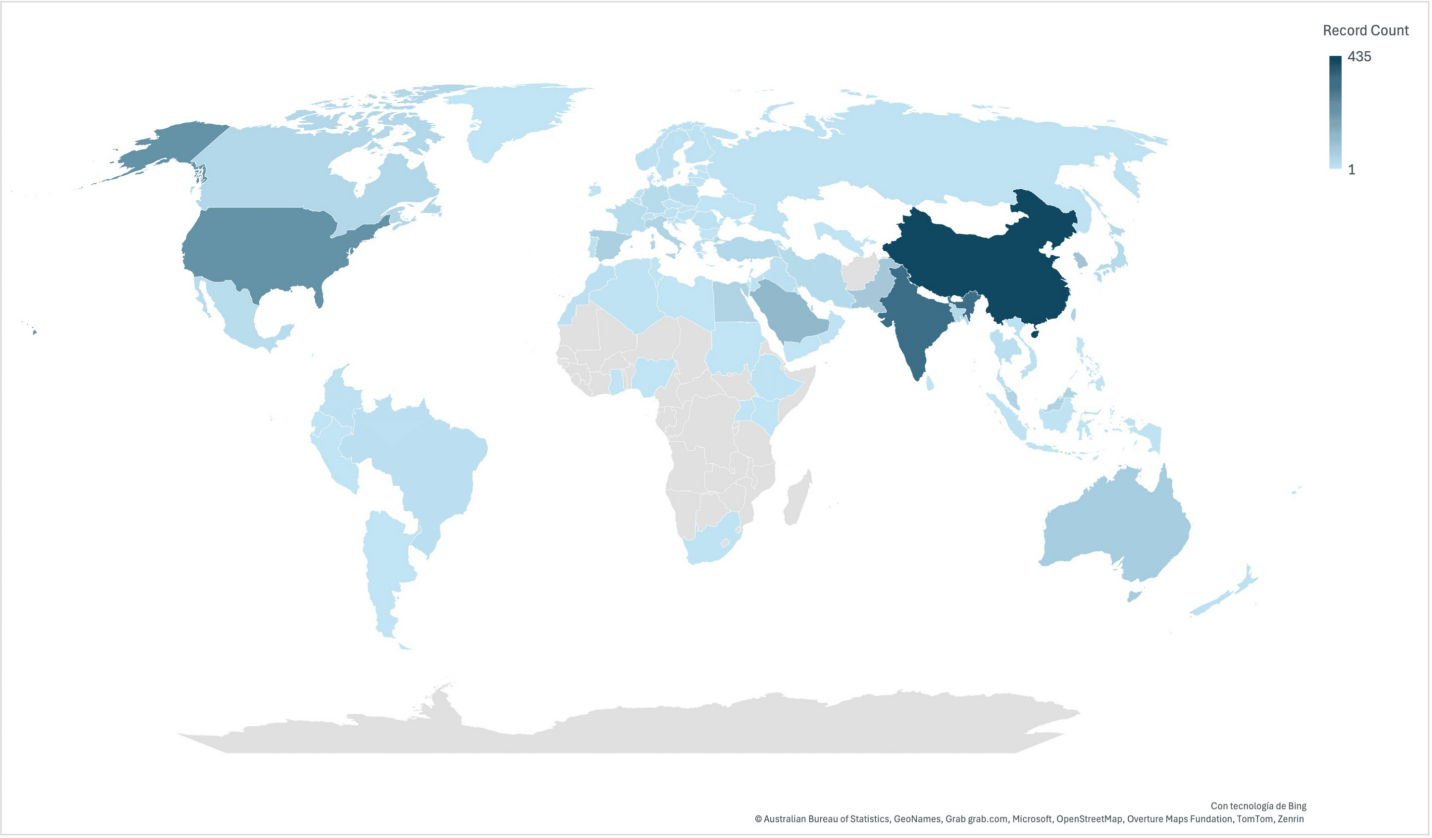
Distribution of the total number of publications by country on artificial intelligence in type 2 diabetes mellitus. The color intensity represents the number of publications, with darker shades indicating a higher number of publications and lighter shades indicating fewer publications. Countries in gray have no records in the analyzed database.

**Figure 5:**
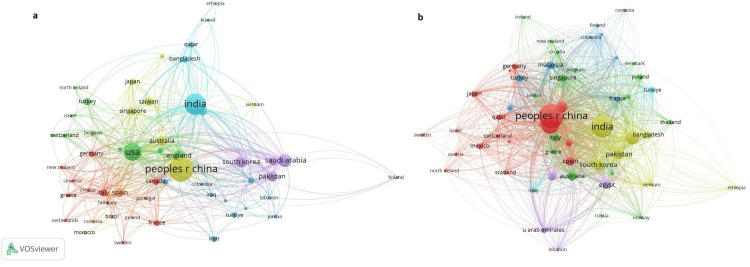
Bibliometric map of country distribution. (a) Cross-country citation analysis network map. (b) Network map of co-authorship analysis between countries. Figure created by the authors using VOSviewer.

A total of 92 countries published articles on the application of AI in the field of T2DM. Table 1 shows the top 10 countries in terms of citation frequency and publication volume based on the corresponding author. The top three countries with the highest number of publications and citations were China (27.39%), India (20.53%), and the United States (14.48%) (Figure 4). These countries also demonstrated stronger links, as seen in the country contribution rankings. India exhibited a collaboration strength of 3,087, surpassing China by 1.42%. The stronger the link between two countries, the thicker the connecting line (Figure 5A). Co-authorship results indicated that China had the most affiliations, linked to 41 countries, with 435 documents and 6,726 citations (Figure 5B).

**Table 1:** Distribution of bibliographic records by 10 main countries. AAC: average article citations; SCP: single country publications; MCP: multiple country publications

Number	Country	Total citations per country		Corresponding author’s country			
		Citations	AAC	Articles	SCP	MCP	MCP ratio
1	China	5,713	13.07	388	365	72	0.1648
2	India	3,551	12.42	278	242	44	0.1538
3	United States	3,491	26.25	133	75	58	0.4361
4	Turkey	1,362	43.94	49	26	5	0.1613
5	United Kingdom	1,335	28.40	49	24	23	0.4894
6	Korea	1,313	17.99	76	40	33	0.4521
7	Singapore	1,105	48.04	25	9	14	0.6087
8	Australia	1,039	30.56	33	15	19	0.5588
9	Greece	818	90.89	9	7	2	0.2222
10	Saudi Arabia	689	10.77	66	25	39	0.6094

Journals and Publications

A total of 463 journals contributed to the research output, with the top 10 most productive journals belonging to six different publishers. Table 2 lists the top 10 journals in terms of the number of publications and their respective TCs. The most productive journal was Institute of Electrical and Electronics Engineers (IEEE Access) (TP = 105 articles, 6.61% of the total), followed by Diagnostics (TP = 55, 3.46%), Sensors (TP = 48, 3.03%), and Applied Sciences-Basel (TP = 46, 2.89%). However, the journals with the highest TC were IEEE Access (TC = 2,345 citations) and Computers in Biology and Applications (TC = 1,004 citations), indicating their relevance in disseminating research on AI in T2DM. Among all, Investigative Ophthalmology & Visual Science, a journal from the Association for Research in Vision and Ophthalmology, received the highest number of citations for a single article at 642 (Table 3).

**Table 2:** Quantitative measurement of research published in journals on artificial intelligence in type 2 diabetes mellitus. TP: total number of articles published on the topic; TC: total number of citations received by the articles; SJR: database reporting the impact metrics of scientific journals; JIF: impact factor of the journals used to measure their quality and visibility; H-Index: a metric that measures both the productivity and citation impact of a researcher’s publications. An H-index of 10 means the researcher has 10 papers with at least 10 citations each; Cites per Doc: the average number of citations per document published in a journal over a defined period; CiteScore: a metric reflecting the average citations per document for a journal each year, calculated over a three-year period

Number	Sources	Country	TP	TC	H-Index	JCI	SJR	Cites per Doc	CiteScore	JIF 2023
1	IEEE Access	United States	105	2,345	242	0.87	0.960	5.183	9.8	3.4
2	Diagnostics	Switzerland	55	487	65	0.87	0.667	3.323	4.7	3.0
3	Sensors	Switzerland	48	996	245	0.87	0.786	4.504	7.3	3.4
4	Applied Sciences-Basel	Switzerland	46	744	130	0.56	0.508	3,135	2.4	2.5
5	Multimedia Tools and Applications	Netherlands	42	633	106		0.801	4.472	3.7	3.0
6	Computers in Biology and Medicine	United Kingdom	33	1,004	125	1.8	1.481	8.757	11.7	7.0
7	Biomedical Signal Processing and Control	Netherlands	32	283	108	1.08	1.284	6.966	9.8	4.9
8	Frontiers in Endocrinology	Switzerland	31	209	120	0.87	1.240	4.367	5.7	3.9
9	Frontiers in Medicine	Switzerland	23	90	86	0.84	0.909	3.359	5.1	3.1
10	Computers Materials & Continua	United States	22	128	57	0.68	0.460	2.895	5.3	2.0

**Table 3:** Top most cited articles on artificial intelligence in type 2 diabetes mellitus. TC: total number of citations received by those articles; TC per Year: total citations per year. It represents the total number of citations an article has received divided by the number of years since its publication. NTC: normalized total citations. It is the raw total of citations received since publication, without normalization

Number	Paper	DOI	TC	TC per Year	NTC
1	Abràmoff MD, 2016, Invest Ophth Vis Sci	10.1167/iovs.16-19964	642	71.3	8.32
2	Kavakiotis I, 2017, Comput Struct Biotec	10.1016/j.csbj.2016.12.005	585	73.1	5.37
3	Zou Q, 2018, Front Genet	10.3389/fgene.2018.00515	346	49.7	7.17
4	Quellec G, 2017, Med Image Anal	10.1016/j.media.2017.04.012	281	35.1	2.58
5	Li JPO, 2021, Prog Retin Eye Res	10.1016/j.preteyeres.2020.1009	274	68.5	12.14
6	Quellec G, 2008, IEEE T Med Imaging	10.1109/TMI.2008.920619	244	14.4	2.48
7	Qummar S, 2019, IEEE Access	10.1109/ACCESS.2019.2947484	243	40.5	5.61
8	Wan SH, 2018, Comput Electr Eng	10.1016/j.compeleceng.2018.07	235	33.6	4.87
9	Mateen M, 2019, Symmetry-Basel	10.3390/sym11010001	217	36.2	5.01
10	Kahramanli H, 2008, Expert Syst Appl	10.1016/j.eswa.2007.06.004	206	12.1	2.09

Institutions and Networks

**Figure 6:**
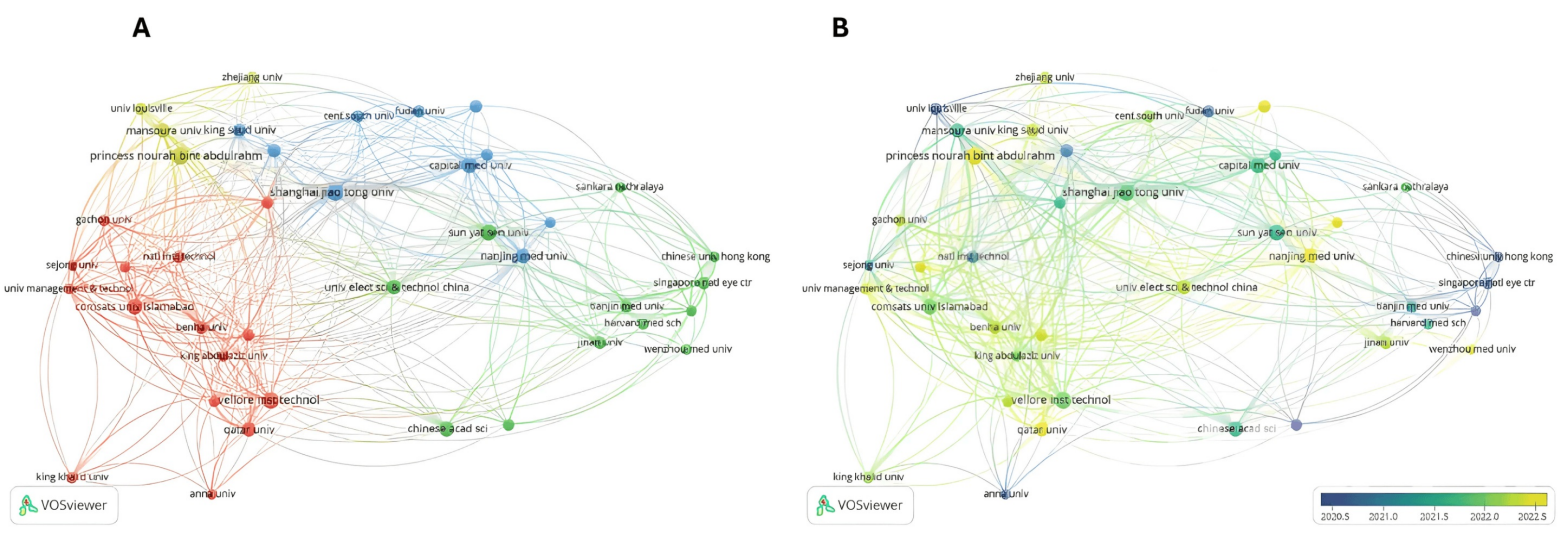
Bibliometric map of the distribution of institutions. A: Network map of collaboration analysis between institutions. B: Overlaid view of the institution over time. The figure was created by the authors using VOSviewer.

The analysis identified 2,482 institutions. Table 4 shows the 15 most productive institutions ranked by citations that have published on AI in T2DM. The network consisted of 41 nodes organized into four different clusters with a publication volume of 10 or more (Figure 6A). Princess Nourah bint Abdulrahman University emerged as the institution with the most publications (TP = 23), followed by VIT Vellore (TP = 22) and Shanghai Jiao Tong University (TP = 22). In terms of citations, Hospital Lariboisière, France (TC = 731), INSERM, France (TC = 614), and Université de Bretagne Occidentale, France (TC = 614) were the top three institutions, with citation frequencies of over 500. Figure 6B shows the timeline of institutional publications; those published earlier are represented in blue tones, while those published later are in yellow tones.

**Table 4:** Top most productive institutions according to the number of citations on artificial intelligence in type 2 diabetes mellitus. TP: total number of articles published on the topic; TC: total number of citations received by those articles; TLS: total link strength Ranking of Top University (https://www.topuniversities.com/world-university-rankings/2024).

Number	Organization	Country	Ranking	TP	TC	TLS - citations	TLS - co-authorship
1	COMSATS University Islamabad	Pakistan	651-660	17	432	173	13
2	Hospital Lariboisière	France	NA	5	731	169	8
3	University of Louisville	United States	1001-1200	12	493	155	18
4	Manchester Metropolitan University	United Kingdom	590	11	478	151	12
5	University of Management and Technology	United States	771-780	10	176	148	14
6	Mansoura University	Egypt	1001-1200	15	298	147	18
7	Qatar University	Qatar	173	16	271	145	21
8	VIT Vellore	India	851-900	22	336	125	9
9	Universiti Kebangsaan Malaysia	Malaysia	159	10	188	111	15
10	Princess Nourah bint Abdulrahman University	Saudi Arabia	661-670	23	142	111	25
11	INSERM	France	NA	7	614	104	10
12	Université de Bretagne Occidentale	France	NA	6	614	102	10
13	King Abdulaziz University	Saudi Arabia	143	10	211	102	6
14	Taif University	Saudi Arabia	NA	9	221	100	11
15	Khulna University	Bangladesh	1201-1400	5	468	99	7

Keywords and Thematic Trends

**Figure 7:**
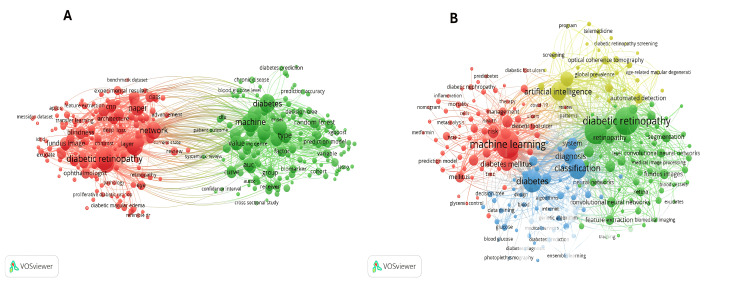
Bibliometric map of keyword distribution. A: Keyword analysis network map. B: Keyword co-occurrence analysis network map. Larger circles reflect more important keywords, lines between terms indicate associations, and thicker lines represent a stronger link between the two terms. The figure was created by the authors using VOSviewer.

Keywords were extracted from titles and abstracts to understand the authors’ perspectives on the topic (Figure 7A). Of the 32,121 terms, 488 meet the minimum criterion. A total of 293 (60%) terms were analyzed using a smaller matching set of 20 occurrences, which was grouped into two clusters. Notable terms included “diabetic retinopathy,” “machine,” and “diabetes,” which were connected to many other words in the network. Based on the co-occurrence visualization for 5,002 keywords, 218 terms (with a minimum occurrence of 10 keywords) were identified and classified into four groups of different colors. Research topics focused on DR detection using ML (Figure 7B). AI and T2DM constitute a multidisciplinary field, but their study is not as fragmented.

Most Cited Articles and Authors

Table 3 presents the 10 most cited articles, 70% of which focus on ophthalmological topics such as DR, while 30% address the use of DL. Among these works, the article titled “Improved Automated Detection of Diabetic Retinopathy on a Publicly Available Dataset Through Integration of Deep Learning,” published in Investigative Ophthalmology & Visual Science in 2016, stood out with the highest number of citations. This study demonstrated significant improvements in DR detection using DL, achieving an area under the curve of 0.980, sensitivity of 96.8%, and specificity of 87% [47].

Of the articles, 2.52% (40) were written by a single author, while the document-to-author ratio was 0.233, and the average number of co-authors per article was 5.55. Among the 7,347 authors, 87 contributed to four articles, with Rajiv Raman being the most prolific author (TP = 13, TC = 135), followed by Ayman El-Baz (TP = 10; TC = 330). Gwenole Quellec (TP = 7), Beatrice Cochener (TP = 6), and Mathieu Lamard (TP = 6) had the highest number of citations (TC = 614).

**Figure 8:**
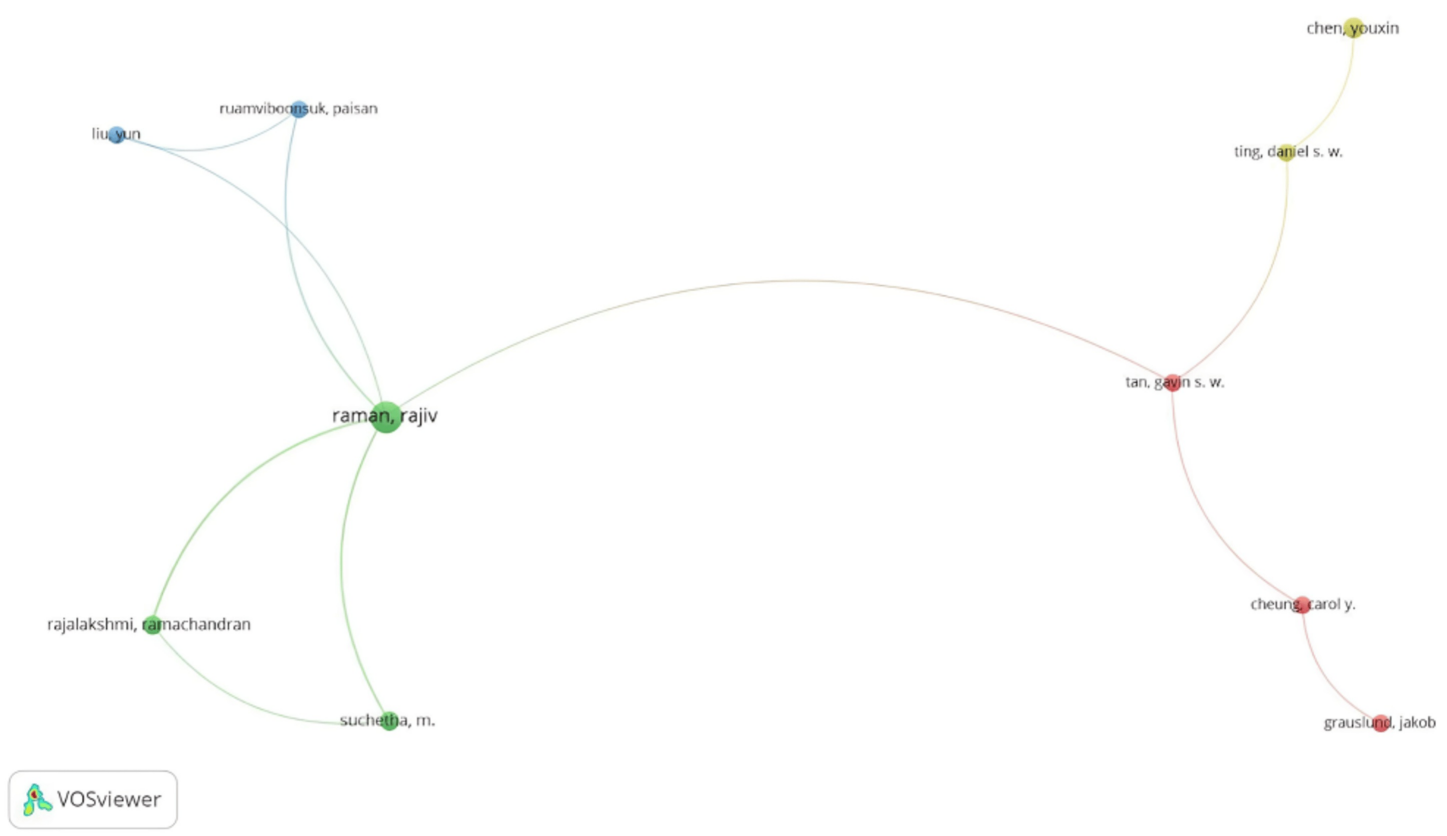
Map of research networks of authors with at least four articles. The figure was created by the authors using VOSviewer.

The co-citation map revealed four clusters of authors, showcasing the interrelationships among all cited authors. Only 11 (0.15%) authors formed a collaboration network (Figure 8). The percentage of international co-authorship was 30.42%. Two clusters consisted of three authors (red and green), while two clusters included two authors each (blue and yellow). The author with the strongest co-authorship was Rajiv Raman, followed by Tan Siew Wei Gavin.

**Figure 9:**
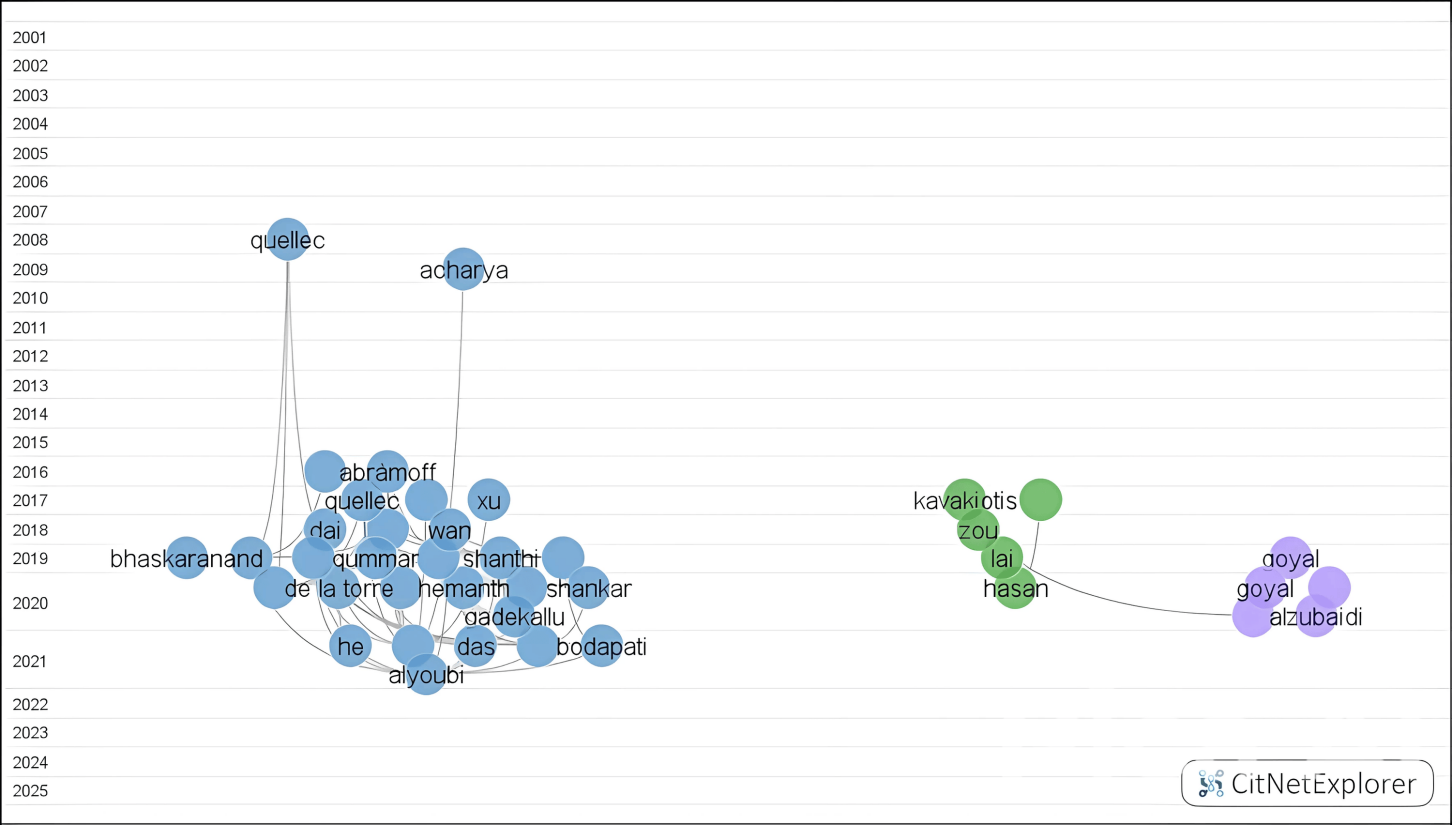
Cluster map based on the citation network. The figure was created by the authors using CitNetExplorer.

Five clusters were identified using the CitNetExplorer clustering technique (Figure 9). During the process, 357 publications were excluded from clusters due to the minimum size requirement (10 publications). The clustering technique classified publications into five groups ranging from 2007 to 2022. Cluster 1 included 661 publications, of which 60 had citation scores above 15. Cluster 2 included 430 publications, of which 16 had citation scores above 15. The percentage of international co-authorship across clusters was 30.42%.

Discussion

The bibliometric analysis of the past two decades revealed exponential growth in research on AI applied to T2DM, with notable acceleration during the 2020-2024 period. This progress reflects not only the increasing relevance of AI in managing T2DM but also its consolidation in other areas of medicine. For instance, in geriatrics, AI research exhibited a similar pattern, with 90.87% of publications occurring between 2014 and 2022, showing a marked increase since 2020 [49]. Similarly, in critical care medicine, the use of AI experienced sustained growth starting in 2018, achieving an annual growth rate of 135.3% that year, with 72.53% of its publications concentrated between 2018 and 2022 [50]. In oncology, the number of publications rose from 235 articles in 2017 to 440 in 2018, reaching an estimated 1,872 publications by 2022 [51]. This increase in publications can be correlated with advancements in telemedicine technologies and the growing availability of digitized healthcare systems that facilitate real-time patient data collection [17]. These trends underscore the convergence of healthcare innovation needs with advancements in AI techniques, particularly in DL algorithms and neural networks. These tools have revolutionized the ability to process large volumes of clinical data efficiently, optimizing both research and medical practice [11,52,53].

Within AI applications for T2DM, the analysis reveals that the most concentrated topics include DR, continuous glucose monitoring, and predictive models for cardiovascular and renal complications. The identification of DR using computer vision algorithms stands out as a key focus due to the high costs and complexity of manual diagnosis [54,55]. Advances in medical imaging technologies and growing awareness of the importance of early detection have driven the generation of large datasets essential for training and validating ML and DL models [56,57]. Consistent with this study, a previous bibliometric analysis showed marked interest in DR starting in 2018, with an average growth rate of 127.02% and 262 publications in 2021 [58].

Real-time glucose monitoring and AI-based artificial pancreas systems that automatically adjust insulin doses using ML algorithms have demonstrated improvements in glycemic control and reduced glucose variability, preventing long-term complications [15,53,59]. In the management category, research included common ML techniques such as logistic regression, random forest, support vector machines, linear regression, and reinforcement learning. Despite lower productivity compared to the prediction category since 2017, this line of research highlights key clinical priorities and funding challenges. As the scientific community gains confidence in the applicability and accuracy of these ML and DL techniques, their use is expected to expand, encompassing both early detection and advanced management. Future applications could include early predictions of glycemic excursions based on continuous monitoring data or reduced hypoglycemic events through improvements in basal-bolus treatments [60]. As these technologies mature, they could integrate into automated insulin delivery systems that leverage ML and DL to analyze large datasets, generate control strategies, and develop complementary models. These systems would facilitate the learning of complex behaviors that are difficult to model analytically [61,62].

Predictive models are also prominent in the literature as a key tool for anticipating renal, cardiac, and neurological complications associated with T2DM. AI algorithms that integrate data from multiple sources, such as medical history, laboratory parameters, and biomarkers, enable advanced risk stratification, facilitating early intervention [63]. Studies have shown that these models help identify patients at higher risk of progressing to severe complications, promoting personalized treatments and optimizing healthcare system resources [18].

International collaborations and institutional co-authorship are essential for research on AI applied to T2DM. The co-authorship analysis showed that China leads in terms of collaborative affiliations, followed by India and the United States. In other areas of AI applied to health, the United States leads in publications on cancer detection, with 1,627 articles, followed by China and India, with 1,202 and 1,079 publications, respectively [51]. In the fields of intensive care and geriatrics, the United States also ranks first, followed by China, with the United Kingdom in third place for intensive care and Japan for geriatrics [49,50]. These countries have established robust scientific collaboration networks, enabling the sharing of infrastructure and specialized knowledge in AI and health [64]. The importance of these collaborations becomes even more evident when considering low- and middle-income countries, which may lack the resources necessary to conduct independent research on AI applied to health. These nations significantly benefit from international partnerships, which increase their access to advanced technologies and enable model validation in more diverse population contexts [19].

The author with the most publications identified in this study was Rajiv Raman, a prominent retinal surgeon and national expert in DR. Raman is recognized as one of India’s most innovative specialists and researchers in retina, with his leadership in the field reflecting the country’s growing prominence in AI research on T2DM [65]. Ayman El-Baz, a PhD in electrical and computer engineering, ranks as the second author with the most publications in AI applied to T2DM. His distinguished career spans areas such as biosignaling, bioimage modeling, and computer-assisted functional diagnostic systems, with significant contributions not only in this field but also in oncology, nephrology, and neurology [66]. Currently, El-Baz serves as the Department Chair and Professor of Bioengineering at the University of Louisville, a position that has significantly influenced the institution’s ranking as the second most prolific in publications on the subject [67].

Other key institutions contributing to scientific production on AI and T2DM include Hospital Lariboisière in France and VIT Vellore in India. These institutions have made significant contributions not only in terms of publication volume but also in the impact of their research. Collaboration between hospitals and universities facilitates a translational approach to research, accelerating the transfer of AI discoveries into clinical practice [67].

In this review, IEEE Access [68] stood out as the leading journal in both production and citations. Its focus is not limited to T2DM but spans other areas of medicine. It is the second most prolific journal for publications on AI in DR, the tenth in cancer detection, and the seventeenth in critical care. This reflects its ability to attract innovative and multidisciplinary research, covering a wide range of AI applications across various areas of medicine.

The analysis of keywords provides valuable guidance for future research and highlights the most relevant aspects of AI applied to T2DM. In this case, the identified keywords were grouped into four main categories. The first group was led by the term “artificial intelligence,” establishing a direct connection with the second group. The second group highlighted “machine learning” as the most utilized AI method. The third group focused on terms related to the disease itself, such as “diabetes,” “classification,” and “diagnosis.” The fourth group addressed complications associated with T2DM, with “diabetic retinopathy” and “retinopathy” being the most representative keywords.

The most cited article was “Improved Automated Detection of Diabetic Retinopathy on a Publicly Available Dataset Through Integration of Deep Learning.” Published in 2016, this article compares the performance of two algorithms for the automated detection of DR, demonstrating that the DL-enhanced algorithm performs significantly better than the one without DL [69]. The high citation count of this article may be attributed to its importance and applicability in clinical practice.

Despite significant advancements, there are important limitations in the application of AI to T2DM that require attention. The availability of accurate and labeled data is one of the main challenges. The quality of AI models heavily depends on the availability of representative and high-quality data, which is not always feasible in the healthcare context [70]. Moreover, bias in datasets can affect the generalizability of algorithms, limiting their applicability across diverse populations [18]. This issue is particularly relevant for AI in T2DM, as the disease exhibits heterogeneity in its manifestation and progression, influenced by factors such as socioeconomic environment and ethnic background [71].

The use of AI in healthcare raises significant ethical concerns, particularly regarding patient data privacy and informed consent. In many countries, regulations governing personal data are strict, which can limit the collection and use of data in AI research. Moreover, AI algorithms relying on sensitive data must be transparent and auditable to avoid automated decisions without medical oversight [18]. The implementation of clear regulatory frameworks and training in data ethics is essential to ensure the safe and ethical adoption of AI in T2DM care [17]. Another major limitation is the lack of model validation across different clinical contexts and populations. Algorithms trained on specific populations may not perform adequately in others due to genetic variations, lifestyles, and socioeconomic factors. This underscores the need for multicenter studies and the inclusion of diverse populations in validation trials [10]. Future research should focus on developing robust and transferable AI models to ensure their effectiveness in populations with distinct demographic characteristics.

AI applied to T2DM presents significant opportunities for future research. One promising area is the use of ML algorithms to integrate multimodal data, including genomic, environmental, and lifestyle information. This could improve understanding of T2DM etiology and offer new opportunities for personalized treatment. Additionally, AI systems have the potential to be integrated into primary healthcare, enabling more accessible and continuous monitoring of T2DM patients, especially in communities with limited access to diabetes specialists [16].

AI in public health could also help identify risk factors in specific populations, facilitating preventive interventions. AI systems could analyze population-level data to detect patterns indicating an elevated risk of T2DM, enabling the implementation of personalized prevention programs. The application of AI in this field requires collaboration between governments and communities to ensure data is collected and used ethically and effectively [72].

Limitations

This analysis was limited to publications indexed in WoS, which may have excluded high-impact articles available in other databases such as Scopus or PubMed. Reliance on a single database can introduce selection biases that affect the generalizability of the findings. Additionally, this analysis did not evaluate the methodological quality of the studies, which would be essential to identify the limitations of AI approaches in T2DM. Future research could expand this analysis to other databases and consider a systematic review of the methodology used in the most cited studies to help identify best practices and specific limitations of AI methods in T2DM [22]. The application of AI in T2DM is transforming the diagnosis and management of this disease, offering innovative solutions ranging from early detection to personalized treatment. International collaboration and interdisciplinarity have been key to the development of this field, enabling the creation of highly specialized and effective AI models. However, the implementation of these technologies faces significant challenges, particularly regarding data quality, patient privacy, and validation across diverse clinical contexts.

## Conclusions

This analysis has identified current trends and future research areas, highlighting the relevance of an ethical and collaborative approach to maximizing the potential of AI in the care of T2DM. A marked increase in scientific output has been observed since 2020, led primarily by China, India, and the United States, countries that also exhibit stronger collaborative ties. Key research areas include electrical and electronic engineering, focused on the development of devices for glucose diagnosis, monitoring, and prediction; and medical disciplines such as endocrinology and ophthalmology, concentrating on studying the natural history of the disease, particularly complications such as macular edema and DR. In conclusion, the consolidation of AI in this field will require ongoing efforts in innovation, regulation, and international collaboration to ensure its benefits are equitably and effectively accessible across different socioeconomic and cultural settings.

## Appendices

Search strategy in Web of Science

Topic 1

TS = [artificial intelligence OR machine intelligence OR robot* OR robot technology OR assistant robot OR robot-assisted OR computational intelligence OR computer reasoning OR deep learning OR computer vision system OR sentiment analysis OR machine learning OR neural network* OR data learning OR expert* system* OR natural language processing OR support vector machine* OR decision tree* OR data mining OR deep learning OR neural network* OR bayesian network* OR intelligent learning OR feature* learning OR feature* extraction OR feature* mining OR feature* selection OR unsupervised clustering OR image* segmentation OR supervised learning OR semantic segmentation OR deep network* OR neural learning OR neural nets model OR graph mining OR data clustering OR big data OR knowledge graph]

Topic 2

TS = [Diabetes Mellitus Type 2 OR Type 2 Diabetes Mellitus OR Type 2 Diabetes OR Diabetes Mellitus Type OR Type II Diabetes Mellitus OR Type II Diabetes]
